# Supplementary material for: Comparison between the persistence of post COVID-19 symptoms on critical patients requiring invasive mechanical ventilation and non-critical patients
Source: PLoS One. 2022 Aug 22;17(8):e0273041. doi: 10.1371/journal.pone.0273041 (PMC9394845; doi:10.1371/journal.pone.0273041)
Supplement: S1 Appendix — Institutional ethics committee approval dictamen. (PDF) [file pone.0273041.s001.pdf]

Comité de Investigación
19 CI 09 012 013
Comité de Ética en Investigación
CONBIOÉTICA-09CEI-003-20160427

Ciudad de México a 27 de mayo de 2021
INER/CI/160/2021
INER/CEI/168/2021

Dr. Miguel Ángel Salazar Lezama
Investigador Principal

Asunto: ***DICTAMEN APROBACIÓN.***

Título del Proyecto: ESPECTRO CLÍNICO DEL COVID-19 POSAGUDO EN PACIENTES DESPUÉS DE LA HOSPITALIZACIÓN: ESTUDIO DE COHORTE.

Código asignado por el Comité: **C22-21**

Le informamos que su proyecto de referencia ha sido evaluado por el Comité y las opiniones acerca de los documentos presentados se encuentran a continuación:

	No. y/o Fecha Versión	Decisión
Protocolo	Versión 1, mayo 2021	APROBADO

Este protocolo tiene vigencia de mayo 2021 a mayo 2022.

En caso de requerir una ampliación, le rogamos tenga en cuenta que deberá enviar al Comité un reporte de progreso al menos 30 días antes de la fecha de término de su vigencia. El Comité dispone en su página electrónica de un formato estándar que podrá usarse al efecto. Lo anterior forma parte de las Consideraciones mínimas para el desarrollo del Proyecto de Investigación las cuales vienen descritas al reverso de esta hoja.

Atentamente

Dra. Blanca Margarita Bazán Perkins
Presidente del Comité de Investigación

Dra. Patricia Gorocica Rosete
Secretaria del Comité de Ética en Investigación

México 2021 Año de la Independencia

SALUD
SECRETARÍA DE SALUD

INER
INSTITUTO NACIONAL
DE ENFERMEDADES
RESPIRATORIAS
ISMAEL COSÍO VILLEGAS

Ciudad de México a 04 de mayo del 2021.

Asunto: **Solicitud Carta de dispensa del consentimiento informado.**

Lic. Adriana Espinosa Jove
Presidente del Comité de Ética en Investigación
Presente.

Solicito la dispensa de consentimiento informado para la realización del protocolo de investigación **"Espectro clínico del COVID-19 posagudo en pacientes después de la hospitalización: Estudio de cohorte."** Lo anterior con base en lo establecido en la Declaración de Helsinki, Fortaleza Brasil 2013, en donde se estipula que, "*para la investigación médica en que se utilice material o datos humanos identificables, como la investigación sobre material o datos contenidos en biobancos o depósitos similares, el responsable del proyecto debe pedir el consentimiento informado para la recolección, almacenamiento y reutilización. Podrá haber situaciones excepcionales en las que será imposible o impracticable obtener el consentimiento para dicha investigación. En esta situación, la investigación sólo puede ser realizada después de ser considerada y aprobada por un comité de ética en investigación*".

El protocolo arriba mencionado es un estudio retrospectivo, se recabarán datos del expediente clínico y la información se mantendrá confidencial a través del uso de códigos alfanuméricos, posteriormente será reportada a nivel grupal. Por motivos de contingencia a los pacientes se les realizará únicamente un cuestionario vía telefónica, motivo por el cual se solicita la presente. Agradezco de antemano las facilidades que se otorguen para la realización de este proyecto.

Instituto Nacional de Enfermedades Respiratorias
Comité de Ética en Investigación
27 MAYO 2021
APROBADO
INER

Atentamente:

Dr. Miguel Ángel Salazar Lezama
Investigador responsable

Calzada de Tlalpan 4502, Col. Sección XVI, CP 14080, Alcaldía Tlalpan,
Ciudad de México Tel. 5554871700, www.iner.salud.gob.mx

México 2021
Año de la Independencia
