# Supplementary material for: Comparison between the persistence of post COVID-19 symptoms on critical patients requiring invasive mechanical ventilation and non-critical patients
Source: PLoS One. 2022 Aug 22;17(8):e0273041. doi: 10.1371/journal.pone.0273041 (PMC9394845; doi:10.1371/journal.pone.0273041)
Supplement: S2 Appendix — (PDF) [file pone.0273041.s002.pdf]

INSTITUTO NACIONAL DE ENFERMEDADES RESPIRATORIAS

ISMAEL COSÍO VILLEGAS

DIRECCIÓN MÉDICA

SUBDIRECCIÓN DE ATENCIÓN MÉDICA NEUMOLÓGICA

OFICINA DE ADMISIÓN HOSPITALARIA Y REGISTROS MÉDICOS

Name of patient:				Age:
Symptom onset date:	Hospital admission date:			
Intubation:	Duration:			
Tracheostomy:	Decanulation:			
Gastrostomy:	Oxygen saturation upon admission:			
Duration of hospitalization:	Post COVID 19 follow-up:			
Comorbidities:	Diabetes		Chronic Obstructive Pulmonary Disease (COPD)	
	Hypertension		Asthma	
	Dyslipidemia		Obstructive Sleep Apnea Syndrome (OSAS)	
	Obesity I		Pulmonary fibrosis	
	Obesity II		Deep vein thrombosis	
	Obesity III		Benign Prostatic Hyperplasia (BPH)	
	Hypothyroidism		Ischemic Heart Disease	
	Glaucoma		Renal Insufficiency	
Risk factors:	Smoking:		Smoking Index:	

From the following signs and symptoms, select with an **X** those that you have presented during the infectious period and after your discharge for COVID 19.

SIGNS AND SYMPTOMS DURING COVID-19

	Dyspnea
	Cough
	Tracheal Stenosis
	Chest pain
	Wheezing
	Palpitations
	Tachycardia
	heart murmurs
	Pericarditis
	Lower limb edema
	Elevated blood pressure
	Fatigue
	Fever
	Cognitive impairments
	Headache
	Cold sensitivity
	Sleep-wake disorders
	Paresthesias/neuralgia
	Lack of sensation
	Vertigo
	Dizziness
	Delirium
	Mobility limitations
	Asthenia
	Adynamia
	Abdominal pain
	Nausea
	Diarrhea
	Constipation
	Lack of appetite
	Arthralgias
	Myalgia
	Depression
	Anxiety
	Tinnitus
	Earache
	Odynophagia
	Hearing loss
	Dysgeusia
	Anosmia
	hyposmia
	Dysosmia
	Rhinorrea
	Sweat
	Skin rash
	Hair loss
	Pressure ulcers
	Muscular weakness
	Tremor
	Abnormalities of vocal cords

POST-COVID 19 SIGNS AND SYMPTOMS

	Dyspnea
	Cough
	Tracheal Stenosis
	Chest pain
	Wheezing
	Palpitations
	Tachycardia
	heart murmur
	Pericarditis
	Lower limb edema
	Elevated blood pressure
	Fatigue
	Fever
	Cognitive impairments
	Headache
	Cold sensitivity
	Sleep-wake disorders
	Paresthesias/neuralgia
	Lack of sensation
	Vertigo
	Dizziness
	Delirium
	Mobility limitations
	Asthenia
	Adynamia
	Abdominal pain
	Nausea
	Diarrhea
	Constipation
	Lack of appetite
	Arthralgias
	Myalgia
	Depression
	Anxiety
	Tinnitus
	Earache
	Odynophagia
	Hearing loss
	Dysgeusia
	Anosmia
	hyposmia
	Dysosmia
	Rhinorrea
	Sweat
	Skin rash
	Hair loss
	Pressure ulcers
	Muscular weakness
	Tremor
	Abnormalities of vocal cords
