# Supplementary material for: Comparison between the persistence of post COVID-19 symptoms on critical patients requiring invasive mechanical ventilation and non-critical patients
Source: PLoS One. 2022 Aug 22;17(8):e0273041. doi: 10.1371/journal.pone.0273041 (PMC9394845; doi:10.1371/journal.pone.0273041)
Supplement: Attachment — Submitted filename: PONE-D-21-28914_reviewer comments.pdf [file pone.0273041.s004.pdf]

The authors followed-up 280 patients that recovered from COVID19 during the period of December 23rd 2020 to April 24th 2021 and reported long-term symptoms associated with COVID19. While this study is important and I would encourage studies like this to be published to get more information on the long-term impact of COVID19 in human health; I would suggest a major revision for this study.

Firstly, I identified a major ethical issue. Study-participants did not sign a consent form even when they went to clinic for evaluation. The authors state that a verbal consent was obtained, but this cannot be verified. Also, data were collected before the authors got an approval from the ethics committee. Good Clinical Practice (GCP) dictates that the study protocol get approval by the institutional review board before any study data are collected or accessed. The researchers need to make sure that their study meets the international ethical standards.

Line 66-68: The percentages are misleading. They refer to certain areas and not the global levels. Needs to be corrected either have percentages that depict the global impact or focus on specific areas of the world.

Line 74: the authors refer to Long-Term COVID-19, please elaborate. Do you mean that these people had COVID-19 infection for long period of time or that the side effects after COVID-19 infection lasted for long time?

Line 93: Please elaborate on the criteria for severe infection. Since these may differ in different parts of the world this need to be defined.

Line 95: For the patients that had the PCR test do you have data on the corona virus strain? If yes it would be interesting to see if these long-term symptoms are associated with certain corona virus strain.

Line 110: Please provide the questionnaire that was used to these patients. Did this questionnaire got approval from the ethics committee before given to the study-participants?

Line 113: Please elaborate on the clinical examination. What did it include? What were the complementary examinations?

Line 116: What was the condition of these patients at baseline before COVID19? Are the symptoms described de novo for these patients?

Line 123: Musculoskeletal symptoms is this due to prolonged hospitalization? Not directly related to COVID19 infection.

Line 127: There is an increase of anxiety and depression cases in global level due to socioeconomical changes that happened during the pandemic. Not directly related to the infection.

Line 166: Oxygen saturation was ranging from 20 to 97%? It is not clear. Did you measure oxygen saturation to only 73.45% of the patients? I am not sure why oxygen saturation hasn’t been reported for all the patients.

Line 171: Only 12.5% of the patients were previously healthy. It would be interesting to see the comparison of the long-term symptoms of COVID19 infection to healthy patients versus the patients with preexisting conditions.

Line 174: I am not sure if this is indeed gender-related. This could be attributed to a high % of men with pre-existing conditions.

Line 193: Gender oriented analysis is needed. Did you see differences in these symptoms between men and women?

Intubated and non-intubated is not the only variable here. I am assuming that the patients that had to be intubated must have had pre-existing conditions, so the differences reported between the two groups could be expected.

What were the treatments these participants received during their hospitalization? Could some of the reported symptoms be attributed to the high-doses of steroid treatments? You could group your patients based on the treatment received and correlate the long-term symptoms after hospitalization.

The authors collected valuable data that could give us an insight on the long-term health impact of COVID19 infection, however its is important to take under consideration the multiple factors that could have contributed to the symptoms described.
