# Supplementary material for: Comparison between the persistence of post COVID-19 symptoms on critical patients requiring invasive mechanical ventilation and non-critical patients
Source: PLoS One. 2022 Aug 22;17(8):e0273041. doi: 10.1371/journal.pone.0273041 (PMC9394845; doi:10.1371/journal.pone.0273041)
Supplement: Attachment — Submitted filename: PONE-D-21-28914_Review.docx [file pone.0273041.s005.docx]

Summary: Irisson-Mora et al. have conducted an interesting study comparing the long-term sequelae of COVID-19 between hospitalized patients who required mechanical ventilation and hospitalized patients that did not. The authors administered a survey to patients with confirmed COVID at 6 months after discharge that captured 8 domains of symptoms and compared the prevalence of symptoms between groups. They found that mechanically ventilated patients, unsurprisingly, had a very high prevalence of persistent symptoms at 6 months (98%), and had higher odds of developing neuropsychiatric and musculoskeletal symptoms that patients that were not mechanically ventilated. The strength of the study is that seems to be well designed with appropriate statistical analysis. The major limitation of the study is the lack of novelty of the results, which may be rectified by some discussion about how the findings in this cohort compare to what is expected in patients who suffer from post intensive care unit syndrome not due to COVID-19.

Major revisions:

1) While there is some novelty in these findings as the time frame is somewhat longer than other studies of the long-haul COVID and more focus on neuro-psychiatric symptoms, the main conclusions of the study are unsurprising, and many of them have been reported previously (PMID: 34308300, PMID: 34308300). A strength of this is that it increases confidence in the findings, but a major limitation is that it does not seem to add new knowledge to aid in prognosis and understanding the pathophysiology of this phenomenon. Including some discussion comparing how the findings of this cohort differ from what others have found may rectify this somewhat, but if there are no major differences in the findings of this cohort and previous studies, it is difficult to see how this contributes anything novel.
2) Additionally, it’s not clear how many of these persistent symptoms are related to the general phenomenon of post-intensive care unit syndrome (PICS), and how many of these symptoms are related specifically to SARS-CoV-2 infection. The authors acknowledge that many of their reported findings are consistent with the general phenomenon of PICS in lines 258-259, but do not expound on this further. Exploring the differences here would be interesting and increase the novelty of the findings, as differences could give some insight into how SARS-CoV-2 infection specifically impacts and modifies PICS.
3) There has been a lot of work into the development of appropriate survey instruments and outcome measures for the study of PICS (PMID: 34025756; PMID: 30600222; PMID: 30600222). While the domains and measures listed by the authors do seem reasonable, it would be helpful to know more detail about how this survey was developed and whether validated metrics and questionnaires were used, and if not, what validation process and testing went into the questionnaire design. Additionally, including the survey instrument with the questions listed in the supplement would be helpful.

Minor revisions:

1) A description of the criteria for pneumopathy (Lines 176 and 261, and Table 1) would be helpful. Are there formal criteria with pulmonary function testing or is this anyone with a history of lung disease?
2) In Figure 3 legend, specifying the methods used to determine significant differences would be helpful, are the p-values listed for single variable or multivariable analysis?
3) The language in the manuscript is clear but line 146 (“we dumped it into a database”) might benefit from different language that better describes the hard work and care that the authors put into the analysis.
