# Supplementary material for: Comparison between the persistence of post COVID-19 symptoms on critical patients requiring invasive mechanical ventilation and non-critical patients
Source: PLoS One. 2022 Aug 22;17(8):e0273041. doi: 10.1371/journal.pone.0273041 (PMC9394845; doi:10.1371/journal.pone.0273041)
Supplement: Attachment — Submitted filename: Response to Reviewers.docx [file pone.0273041.s006.docx]

**Reviewer #1**

**1.-**Firstly, I identified a major ethical issue. Study-participants did not sign a consent form even when they went to clinic for evaluation. The authors state that a verbal consent was obtained, but this cannot be verified. Also, data were collected before the authors got an approval from the ethics committee. Good Clinical Practice (GCP) dictates that the study protocol get approval by the institutional review board before any study data are collected or accessed. The researchers need to make sure that their study meets the international ethical standards.

**A:** We believe this is a misunderstanding.

All patients discharged from the hospital with COVID-19 diagnosis were followed up by a telephone call, that’s why we described a cohort study. Since patients come from all around the country and due mostly to economic issues, it is impossible for the majority of them to return to this hospital, so the post-COVID clinic calls patients in order to apply a questionary (developed by physicians in this area, not a validated test) to all of discharged ones. During this call, verbal consent is obtained, in order to apply this questionnaire, and if they had any persistent symptoms or associated pathology that requires follow up, they are asked to come back to the hospital, where physical exam and if required, other tests are performed. All data, are collected in the physical chart and electronic data system of our hospital.

Our study consisted in a chart and electronic review (descriptive, transversal and observational), with approval of the Institution Ethics committee (C22-21) before any data was collected. No consent form was required for this kind of study.

Changes have been made in the design and methods section in order to clarify this point.

**2.-**Line 66-68: The percentages are misleading. They refer to certain areas and not the global levels. Needs to be corrected either have percentages that depict the global impact or focus on specific areas of the world.

**A:** The risk of serious illness reported in countries such as China and the United States varies from 12.6 to 23.5%, and the rate of invasive mechanical ventilation (IMV) among these patients ranges 68 from 29.1 to 89.9%.

Changes have been made to the introduction section to clarify this point.

**3.-** Line 74: the authors refer to Long-Term COVID-19, please elaborate. Do you mean that these people had COVID-19 infection for long period of time or that the side effects after COVID-19 infection lasted for long time?

**A:** The term prolonged post-COVID-19 describes persistent symptoms following hospitalization from COVID-19 illness. All of our study patients already had a negative polymerase chain reaction (PCR) test for SARS COV2.

Changes have been made to the introduction section to clarify this point.

**4.-**Line 93: Please elaborate on the criteria for severe infection. Since these may differ in different parts of the world this need to be defined.

**A:** Criteria for severe disease were those defined by the WHO (SpO 2 <94%, ratio of arterial partial pressure of oxygen to fractional inspired oxygen (PaO 2 /FiO 2 ) <300 mmHg, respiratory rate >30 breaths/min or pulmonary infiltrates >50%).

Changes have been made in the design and methods section in order to clarify this point.

**5.-**Line 95: For the patients that had the PCR test do you have data on the corona virus strain? If yes it would be interesting to see if these long-term symptoms are associated with certain corona virus strain.

Please provide the questionnaire that was used to these patients. Did this questionnaire got approval from the ethics committee before given to the study-participants?

**A:** The determination of the COVID-19 variant which patients were positive to was not performed. Changes have been made in the design and methods section in order to clarify this point.

As mentioned before, the questionnaire used in these patients was the one used routinely within the Institute, which was also included in the protocol approved by the ethics committee.

The questionnaire was included in complements.

**6.-**Line 113: Please elaborate on the clinical examination. What did it include? What were the complementary examinations?

**A:** Patients who attended the medical appointment in person underwent to a complete physical examination with special attention to the symptoms reported by the patient. The complementary examinations included laboratory tests and chest tomography based on the clinical findings evidenced during the examination.

Changes have been made in the design and methods section in order to clarify this point.

**7.-**Line 116: What was the condition of these patients at baseline before COVID19? Are the symptoms described de novo for these patients?

**A:** Only new symptoms reported after the patient's discharge were taken into consideration and included in the study.

**8.-** Line 123: Musculoskeletal symptoms is this due to prolonged hospitalization? Not directly related to COVID19 infection.

**A:** When performing the univariate and multivariable analysis between musculoskeletal symptoms and prolonged hospitalization time no statistically significant association was demonstrated (P=0.796), so they were directly attributed to COVID-19 infection.

**9.-** Line 127: There is an increase of anxiety and depression cases in global level due to socioeconomical changes that happened during the pandemic. Not directly related to the infection.

**A:** It is indisputable that these symptoms can be a hard to tell apart if whether they can be caused directly by COVID-19 or by the pandemic socioeconomical factors directly. However, it is important to emphasize that in our study they were attributed to post-COVID-19 symptoms, as they were referred by patients as post-hospitalization symptoms, being consistent with the current concept of prolonged COVID-19, so it is appropriate to define them within the spectrum of symptoms related to COVID-19.

**10.-** Line 166: Oxygen saturation was ranging from 20 to 97%? It is not clear. Did you measure oxygen saturation to only 73.45% of the patients? I am not sure why oxygen saturation hasn’t been reported for all the patients.

**A:** The percentage of 73.45% refers to the mean oxygen saturation measured by pulse oximetry and not to the number of patients. Changes have been made in the results section to clarify this point.

**11.-** Line 171: Only 12.5% of the patients were previously healthy. It would be interesting to see the comparison of the long-term symptoms of COVID19 infection to healthy patients versus the patients with preexisting conditions.

**A:** When performing the univariable and multivariable analysis among healthy patients against patients with previously diagnosed comorbidities, it was concluded that patients with comorbidities reported more respiratory (P=0 .037) and musculoskeletal (P=0.034) symptoms. Changes have been made in the results section to clarify this point.

**12.-**Line 174: I am not sure if this is indeed gender-related. This could be attributed to a high % of men with pre-existing conditions.

**A:** Although men had a greater tendency to be intubated (P=0.016), longer hospitalization time (P=0.028) and more comorbidities (P=0.031), no statistically significant differences were found in relation to any pre-existing comorbidity.

Changes have been made in the results section to clarify this point.

**13.-**Line 193: Gender oriented analysis is needed. Did you see differences in these symptoms between men and women?

Intubated and non-intubated is not the only variable here. I am assuming that the patients that had to be intubated must have had pre-existing conditions, so the differences reported between the two groups could be expected.

What were the treatments these participants received during their hospitalization? Could some of the reported symptoms be attributed to the high-doses of steroid treatments? You could group your patients based on the treatment received and correlate the long-term symptoms after hospitalization.

**A:** When performing the univariate analysis by gender, it was found that women had a greater exposure to biomass (24.2 vs 9.9%, P=0.002), higher prevalence of respiratory symptoms (71.9 vs 59.2, P=0.032), and dermatological symptoms (25.8 vs 9.2%, P=0 .000) compared to men.

Changes have been made in the results section to clarify this point.

We do not have data regarding the treatment received during patients’ hospitalization and its relation with long-term symptoms after COVID-19 infection, therefore they were not included in this study, which represents a limitation of our study and their inclusion would represent the objective from another job.

**Reviewer #2**

**Major revisions:**

**1.-**While there is some novelty in these findings as the time frame is somewhat longer than other studies of the long-haul COVID and more focus on neuro-psychiatric symptoms, the main conclusions of the study are unsurprising, and many of them have been reported previously (PMID: 34308300, PMID: 34308300). A strength of this is that it increases confidence in the findings, but a major limitation is that it does not seem to add new knowledge to aid in prognosis and understanding the pathophysiology of this phenomenon. Including some discussion comparing how the findings of this cohort differ from what others have found may rectify this somewhat, but if there are no major differences in the findings of this cohort and previous studies, it is difficult to see how this contributes anything novel.

**A:** Thank you for your comment, as you have mentioned, there are other longer studies evaluating the prevalence of COVID-19. However, most of these include patients from community settings, with mild or moderate COVID. The novelty of our study is that, as a reference center, we have a significant number of critically ill patients, a population in which long-term sequelae have not been sufficiently explored.

**2.-** It’s not clear how many of these persistent symptoms are related to the general phenomenon of post-intensive care unit syndrome (PICS), and how many of these symptoms are related specifically to SARS-CoV-2 infection. The authors acknowledge that many of their reported findings are consistent with the general phenomenon of PICS in lines 258-259, but do not expound on this further. Exploring the differences here would be interesting and increase the novelty of the findings, as differences could give some insight into how SARS-CoV-2 infection specifically impacts and modifies PICS.

**A:** COVID-19 critical patients may be predisposed to have a greater prevalence of PICS because of longer periods of mechanical ventilation, sedation and the use of steroids and sedative meditation for their treatment. Although PICS is known to be a common syndrome in patients that needed critical care, and there are a significant number of patients that required this kind of care due to COVID, the prevalence of PICS and its definition in patients with COVID is still not yet determined. Such is the case that in 2021 a call has been made to the World Health Organization (WHO) to develop International Classification of Disease Diagnostic Codes for PICS in the “Age of COVID-19” that incentive to care coordination in outpatient settings and to an effective post-acute treatment.

In the discussion section we have expanded the explanation underlying the mechanisms that may predispose critically ill COVID-19 patients to the persistence of the symptoms we found.

**3.-** There has been a lot of work into the development of appropriate survey instruments and outcome measures for the study of PICS (PMID: 34025756; PMID: 30600222; PMID: 30600222). While the domains and measures listed by the authors do seem reasonable, it would be helpful to know more detail about how this survey was developed and whether validated metrics and questionnaires were used, and if not, what validation process and testing went into the questionnaire design. Additionally, including the survey instrument with the questions listed in the supplement would be helpful.

**A:** The questionnaire used in these patients is the one that has been taken out routinely within the Institute since the start of the pandemic, and is included in the protocol approved by the ethics committee for inclusion in this study, available in supplements.

The domains captured in this tool were categorized into the following 8 symptom classes, depending on the organ system affected: [1] Respiratory symptoms, including dyspnea, chest pain, cough, sore throat, rhinorrhea, wheezing, vocal cord abnormalities ( hoarseness, chordal paralysis) and tracheal stenosis. [2] Cardiovascular symptoms such as clubbing, rhythm disturbances (palpitations, tachycardia, and bradycardia), heart murmur, pericarditis, elevated blood pressure, extremity edema, and deep inferior vein thrombosis. [3] Systemic symptoms, such as fever, dizziness, fatigue, asthenia, adynamia, sweating, pruritus, sensitivity to cold, and tremors. [4]. Musculoskeletal symptoms, such as paresthesias, mobility limitations, arthralgias, tendon injuries, myalgias, paresthesia, neuralgia, muscle weakness, low back pain and contractures. [5] Dermatologic symptoms, including hair loss, skin rashes, and pressure ulcers. [6] Neurological and neuropsychiatric symptoms, such as headache, sleep and wake disorders, cognitive disturbances such as poor concentration and memory loss, as well as anxiety disorders and/or depression. [7] Gastrointestinal symptoms, including nausea, diarrhea, constipation, abdominal pain, and lack of appetite. [8] Audiological symptoms, including tinnitus, ear pain, hearing loss, and vertigo. The impact of each domain on the participants' daily function was reported as present or absent.

**Minor revisions:**

**1.-** A description of the criteria for pneumopathy (Lines 176 and 261, and Table 1) would be helpful. Are there formal criteria with pulmonary function testing or is this anyone with a history of lung disease?

**A:** Included comorbidities were those previously diseases documented in the chart and self-reported by patients; pneumopathy was defined as a history of asthma, chronic obstructive pulmonary disease (COPD) and pulmonary fibrosis.

**2.-** In Figure 3 legend, specifying the methods used to determine significant differences would be helpful, are the p-values listed for single variable or multivariable analysis?

**A:** A univariate association analysis was performed using the Chi-square test or Fisher's exact test considering significant P values those less than 0.05. comparing intubated and non-intubated patients.

Changes have been made in the Figure legend to clarify this point.

**3.-** The language in the manuscript is clear but line 146 (“we dumped it into a database”) might benefit from different language that better describes the hard work and care that the authors put into the analysis.

**A:** The word “we dumped” was changed to “were collected on an Excel page”.

Changes have been made in the design and methods section in order to clarify this point.

**Reviewer #3**

**Major comments:**

**1.-** It is not clear from the manuscript the timing of reported symptoms (e.g. in page 16 you state "at of evaluation")- are they all present at 6 months? Were they reported at 2 weeks but dissipated by 6 months? Something in between? Assuming some patients reported symptoms at 3 weeks and other at 5 months and everything in between, this lack of temporal resolution leads to less specific findings, which end up being less clinically meaningful. As a physician, I would not know how to use these results to advise patients- will the symptoms that they have reported 2 weeks after discharge persist for much longer? If they don't have symptoms now, will they develop them later? I couldn't tell. Is there a difference in time course depending on what symptom was reported (eg do PTSD last longer than the paresthesias?). I would try to be more specific about time course.

**A:** We carried out a transversal study including persistent symptoms in the period of time that they attended the first medical evaluation after hospital discharge and that were present up to six months after discharge, these symptoms were included within the definition Late sequelae of SARS CoV-2 infection by lasting more than 4 weeks after initial infection or diagnosis, being consistent with the concept of prolonged COVID.

**2.-** In addition, given the nature of the measured outcome, I would have used time to event analysis (eg time to symptom resolution) to compare those who received IMV vs. not. I would also include "symptom free days" for chosen symptoms in the analysis for an evaluation with less time and death related confounding. If this is done, please add a time to event graph to the figures as this would be more informative than binary bar graphs.

**A:** Being our study a transversal study, we are not measuring the time from the event to the resolution of the symptoms. It would be interesting to carry out another study looking for the differences between the patients. However, its inclusion would represent the object of another study.

**3.-** If possible, please state whether there is a difference in baseline characteristics between patients excluded from the study vs. those who were included.

**A:** For logistical reasons, baseline characteristics among patients excluded from the study were not included.

**4.-** In page 9, when there is a statement about whether IMV was affected by smoking and other co-morbities, please realize that there are several more nuanced analyses in the literature that report that smoking, age, obesity and other comorbidities indeed are associated with higher odds of intubation. These analyses included multivariable analysis while the statement made in this manuscript were evaluated by chi-square only. I would temper the statements re: conclusions from table 1 given the limited analysis provided here.

**A:** When performing the univariable and multivariable analysis regarding comorbidities (P=.467) and risk factors such as smoking (P=.454), biomass exposure (P=.130) and alcoholism (P=.155) comparing intubated patients with non-intubated patients, no statistical significance was found.

**5.-** There are some claims in the discussion that lack sufficient evidence from the results that I would therefore recommend rephrasing or removing. For example, in page 20, the statements made in lines 313-318 lack any evidence from the result section and lack any citations. I would remove this paragraph or at least significantly shorten it. Other paragraphs utilize several lines reviewing the literature without direct connection to the contents of the manuscript and should also be shortened or removed.

**A:** Thank you for your comments. Added quotes from statements in discussion and reworded paragraph have been made.

**Minor comments:**

**1.-** There are several grammatical and lexicon mistakes that need to be corrected- a repeat issue is the use of "sequels" instead of the correct term, "sequelae". In page, 7, I would avoid the use of the colloquialism "dumped" into a scientific publication. Likewise, in page 20, the phrase "the hole COVID hospitalization picture" (lines 314-315) has a typo and is not specific enough for a scientific journal.

**A:** The corresponding grammatical corrections were made, from “sequels” to “sequelae”.

The word "we dumped" was changed to “were collected on an Excel page".

**2.-** Please add error bars to all bar graphs.

**A:** We added error bars to all charts.

**3.-** Regarding symptoms reported, it would be useful to have a table (can be in supplemental materials) defining the symptoms reported- eg, pneumopathy, fatigue, sweat, diarrhea, constipation, cognitive impairments. Were these all subjectively reported or were there an objective measure for some of them? For neuropathic pain, was there a correlation with placement of A line or other procedures? was it generalized neuropathic pain?

**A:** All signs and symptoms included were reported subjectively by the patients, the only objective measure being the physical examination performed during the medical evaluation.

**4.** Likewise, please define risk factors such as smoking history (minimum packs a day? ever or never smoker?), biomass exposure, obesity, and past medical history (were they just extracted from the chart? reported by patients?).

**A:** Risk factors such as smoking, exposure to biomass and alcoholism were reported as recorded in the patient's file and reported as present or absent.

**5.** In line 326-327, usually "tidal volume minimization", which I assume to mean protective mechanical ventilation, will actually decrease oxygenation with the benefit of improved mortality. Decreasing tidal volume will prevent alveolar damage, protect barrier function and mitigate the local release of pro-inflammatory cytokines, but it usually does not improve oxygenation. Please rephrase.

**A:** Some suggested acute-phase interventions for neurological protection include tidal volume minimization in ventilation setting strategies to improve cerebral blood flow and lower cerebral vascular resistance along with less inflammation.

The statement about minimizing tidal volume as a maneuver to improve mortality has been rephrased in the discussion.

**6.-** There is an important point in the discussion re: better outcomes in patients who receive physical therapy (PT). If available, it would be very insightful to report how many patients received PT in the ICU. A comparison in outcomes between those receiving PT v not would be very interesting. A statement comparing the percentage of patients with COVID who received PT vs. historical numbers pre pandemic would be enough to highlight the issues re:access to standard ICU care in this population.

**A:** Although it would be very enriching to include variables on in-hospital management, including physiotherapy during hospitalization, this study did not include this information since its objective was to describe persistent symptoms after hospital discharge and not the effect of interventions during hospitalization.

**7.-** In the conclusion, or maybe in the discussion, one could also mention the likely importance of lighter sedation goals to outcomes, and cite the appropriate sources.

**A:** The use of some sedative agents such as dexmedetomidine instead of benzodiazepines, less use of continuous deep sedation, along with and a conservative fluid management have also been proposed as useful interventions to maximize neurological function.

In the discussion section we have expanded the explanation to clarify this point.
