# Supplementary material for: Comparison between the persistence of post COVID-19 symptoms on critical patients requiring invasive mechanical ventilation and non-critical patients
Source: PLoS One. 2022 Aug 22;17(8):e0273041. doi: 10.1371/journal.pone.0273041 (PMC9394845; doi:10.1371/journal.pone.0273041)
Supplement: Attachment — Submitted filename: Response to Reviewers.docx [file pone.0273041.s007.docx]

We welcome comments raised by reviewers. The limitations of this study have already been added in the discussion section (line 363-368).

The list of references was checked. The reference previously cited as number 42 (PMID: 15489373) has been replaced by current relevant references. Reference number 11 (PMID: 34653099) was added to provide a better definition of the post-COVID-19 syndrome, and reference number 17 was attached to explain the criteria used to define serious illness, as proposed by Reviewer #1 (comment number 3 and 4). References listed as 20-22 (PMID: 3225038, PMID: 32678530, PMID: 33821196) were included for expanded explanation underlying the mechanisms that may predispose critically ill COVID-19 patients to present persistent symptoms and develop the Post-Intensive Care unit Syndrome (PICS). With the intention of delving into the concept of neuropsychiatric manifestations, the references listed as 46-48 (PMID: 34491282, PMID: 33483692, PMID: 30229570) were added. To deeply describe some suggested acute-phase interventions for neurological protection, references listed as 49, 50, 52, and 53 (PMID: 35035429, PMID: 10793162, PMID: 25111642, PMID: 22859526) were added, as suggested by reviewer number 3.
